# Supplementary material for: Evaluation of cutaneous immune response in a controlled human in vivo model of mosquito bites
Source: Nat Commun. 2022 Nov 17;13:7036. doi: 10.1038/s41467-022-34534-9 (PMC9672097; doi:10.1038/s41467-022-34534-9)
Supplement: Supplementary file 1 — Supplementary Information [file 41467_2022_34534_MOESM1_ESM.pdf]

SUPPLEMENTARY INFORMATION

Supplementary Tables

Characteristic	Total	Immunohistochemistry	RNASeq	Flow Cytometry
n	30	10	10	10
Age, median years [IQR]	32.5 [21.75, 37]	33.5 [22.0, 35.0]	33.5 [25.5, 38.5]	30.0 [21.0, 36.0]
Female	13 (43.3)	5 (50.0)	5 (50.0)	3 (30.0)
Occupation				
Businessman/seller/ entrepreneur	3 (10.0)	0 (0.0)	0 (0.0)	3 (30.0)
Government Employee	1 (3.3)	0 (0.0)	0 (0.0)	1 (10.0)
Factory Worker	1 (3.3)	0 (0.0)	1 (10.0)	0 (0.0)
Farmer/Agriculture	1 (3.3)	0 (0.0)	1 (10.0)	0 (0.0)
Freelancer	3 (10.0)	0 (0.0)	0 (0.0)	3 (30.0)
Public Company Employee/State Enterprise	4 (13.3)	0 (0.0)	2 (20.0)	2 (20.0)
Soldier	1 (3.3)	1 (10.0)	0 (0.0)	0 (0.0)
Student	2 (6.7)	0 (0.0)	2 (20.0)	0 (0.0)
Unemployed	14 (46.7)	9 (90.0)	4 (40.0)	1 (10.0)
Housing				
House	20 (66.7)	10 (100.0)	10 (100.0)	0 (0.0)
Apartment	10 (33.3)	0 (0.0)	0 (0.0)	10 (100.0)
Socioeconomic Class				
Lower	5 (16.7)	3 (30.0)	0 (0.0)	2 (20.0)
Middle	25 (83.3)	7 (70.0)	10 (100.0)	8 (80.0)
Num. Children in Household				
1-2	13 (43.3)	6 (60.0)	4 (40.0)	3 (30.0)
3-4	13 (43.3)	3 (30.0)	5 (50.0)	5 (50.0)
5+	4 (13.3)	1 (10.0)	1 (10.0)	2 (20.0)

Num. Domestic Water Containers in Home				
1-2	22 (73.3)	7 (70.0)	8 (80.0)	7 (70.0)
3-4	7 (23.3)	3 (30.0)	2 (20.0)	2 (20.0)
5+	1 (3.3)	0 (0.0)	0 (0.0)	1 (10.0)
Frequency of Bednet Use				
Never	9 (30.0)	3 (30.0)	2 (20.0)	4 (40.0)
Rarely	0 (0.0)	0 (0.0)	0 (0.0)	0 (0.0)
Regularly/Most of the Time	4 (13.3)	0 (0.0)	0 (0.0)	4 (40.0)
All of the time	17 (56.7)	7 (70.0)	8 (80.0)	2 (20.0)
Larvicide Use in Home				
No	28 (93.3)	10 (100.0)	10 (100.0)	8 (80.0)
Yes	2 (6.6)	0 (0.0)	0 (0.0)	2 (20.0)
Insecticide Use in Home				
No	19 (63.3)	6 (60.0)	5 (50.0)	8 (80.0)
Yes	11 (36.7)	4 (40.0)	5 (50.0)	2 (20.0)
Mosquito Coil Use in Home				
No	10 (33.3)	7 (70.0)	2 (20.0)	1 (10.0)
Yes	20 (66.7)	3 (30.0)	8 (80.0)	9 (90.0)
Ever Had Dengue Infection				
No	27 (90.0)	9 (90.0)	10 (100.0)	8 (80.0)
Yes	3 (10.0)	1 (10.0)	0 (0.0)	2 (20.0)
Insect Bites in Last 30 Days				
No	2 (6.7)	0 (0.0)	1 (10.0)	1 (10.0)
Yes	28 (93.3)	10 (100.0)	9 (90.0)	9 (90.0)
Mean Num. Bites (Min, Max)	5.9 (3, 10)	4.9 (3, 7)	7.1 (5, 10)	5.6 (3, 8)
Redness				

No	7 (23.3)	0 (0.0)	6 (60.0)	1 (10.0)
Yes	23 (76.7)	10 (100.0)	4 (40.0)	9 (90.0)
Swelling				
No	1 (3.3)	0 (0.0)	1 (10.0)	0 (0.0)
Yes	29 (96.7)	10 (100.0)	9 (90.0)	10 (100.0)
Mean Bite Size, mm (S.D.)				
15 min	5.0 (2.8)	4.4 (1.2)	6.2 (4.1)	4.4 (2.3)
30 min	4.8 (3.3)	4.1 (1.3)	6.0 (4.8)	4.3 (2.7)
4 hours	2.2 (1.2)	1.9 (1.1)	2.6 (0.9)	2.0 (1.5)
48 hours	1.1 (1.2)	1.6 (1.4)	1.3 (1.1)	0.5 (0.7)
Mean OD (S.D.) to *Aedes aegypti* SGE				
Day 0	0.16 (0.08)	0.15 (0.06)	0.17 (0.10)	0.16 (0.07)
Day 14	0.17 (0.07)	0.15 (0.06)	0.17 (0.10)	0.17 (0.06)
Positive on PanBio® Dengue Indirect IgG	30 (100)	10 (100)	10 (100)	10 (100)

Supplementary Table 1. **Cohort Demographics by Sample Evaluation Modality**. All data presented as n (%) unless otherwise stated. OD = optical density SGE = salivary gland extract.

Fluorochrome	Innate 1	Innate 2	Adaptive
**BUV395**	CD3 (BD biosciences, cat# 564001, Clone SK7, Lot#0030938)		CD3 (BD biosciences, cat# 564001, Clone SK7, Lot#0030938)
**BUV496**	CD4 (BD biosciences, cat# 612936, Clone SK3, Lot#0346317)		CD4 (BD biosciences, cat# 612936, Clone SK3, Lot#0346317)
**BUV737**		CD16 (BD biosciences, cat# 612787, Clone 3G8, Lot#1198885)	CCR7 (CD197) (BD biosciences, cat# 749676, Clone 2-L1-A, Lot#1025804)
**BV421**		CD163 (BioLegend, cat# 333611, Clone GHI/61, Lot#B315613)	CD45RA (BioLegend, cat# 304130, Clone HI100, Lot#B333212)
**BV605**		CD14 (BioLegend, cat# 367125, Clone 63D3, Lot#B307957)	CLA (BD biosciences, cat# 563960, Clone HECA-452, Lot#0177315)
**BV650**	CD1c (BioLegend, cat# 331541, Clone L161, Lot#B297623)		CD194 (BD biosciences, cat# 744140, Clone 1G1, Lot#0239874)
**BV711**		CD56 (NCAM) (BioLegend, cat# 331541, Clone 5.1H11, Lot#B318236)	CD183 (CXCR3) (BioLegend, cat# 353732, Clone G025H7, Lot#B264427)
**BV785**	CD69 (BioLegend, cat# 310932, Clone FN50, Lot#B326498)	CD69 (BioLegend, cat# 310932, Clone FN50, Lot#B326498)	CD69 (BioLegend, cat# 310932, Clone FN50, Lot#B326498)
**BB515**		CD117 (BD biosciences, cat# 565172, Clone 104D2, Lot#0119993)	
**PE-Texas Red**	CD45 (BioLegend, cat# 982308, Clone HI30, Lot#B350011)	CD45 (BioLegend, cat# 982308, Clone HI30, Lot#B350011)	CD45 (BioLegend, cat# 982308, Clone HI30, Lot#B350011)
**PE**	CD207 (Langerin) (BioLegend, cat# 352203, Clone 10E2, Lot#B253018)		
**BB700**		CD11b (BD biosciences, cat#	

		746004, Clone D12, Lot#0239883)	
**PE-Cy7**			CD279 (PD-1) (BD biosciences, cat# 561272, Clone EH12.1, Lot#0022949)
**APC**			
**APC-R700**	CD25 (BD biosciences, cat# 565106, Clone 2A3, Lot#0238004)	CD25 (BD biosciences, cat# 565106, Clone 2A3, Lot#0238004)	CD25 (BD biosciences, cat# 565106, Clone 2A3, Lot#0238004)
**APC-H7**	CD8 (BD biosciences, cat# 560179, Clone SK1, Lot#1039448)		CD8 (BD biosciences, cat# 560179, Clone SK1, Lot#1039448)
**Amcyan**	**Viability**	**Viability**	**Viability**

Supplementary Table 2**. List of antibodies used for flow cytometry analysis, clones, catalog numbers, lot numbers and fluorochromes.**

Supplementary Figures

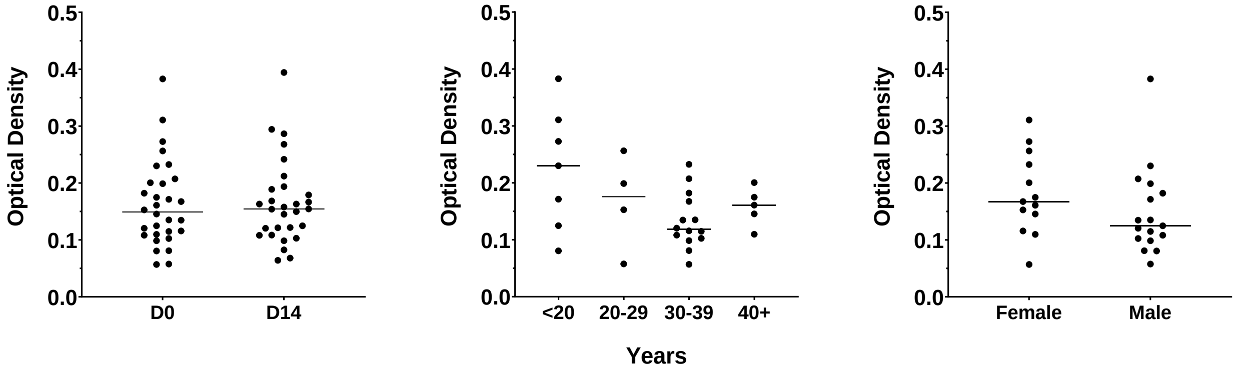

Supplementary Figure 1. **Change in optical density of anti-SGE IgG by time, age, and gender as measured by *Aedes aegypti* salivary IgG ELISA.** No significant differences were noted in the groups. Line represents median. N=30 individuals. Source data are provided as a Source Data file.

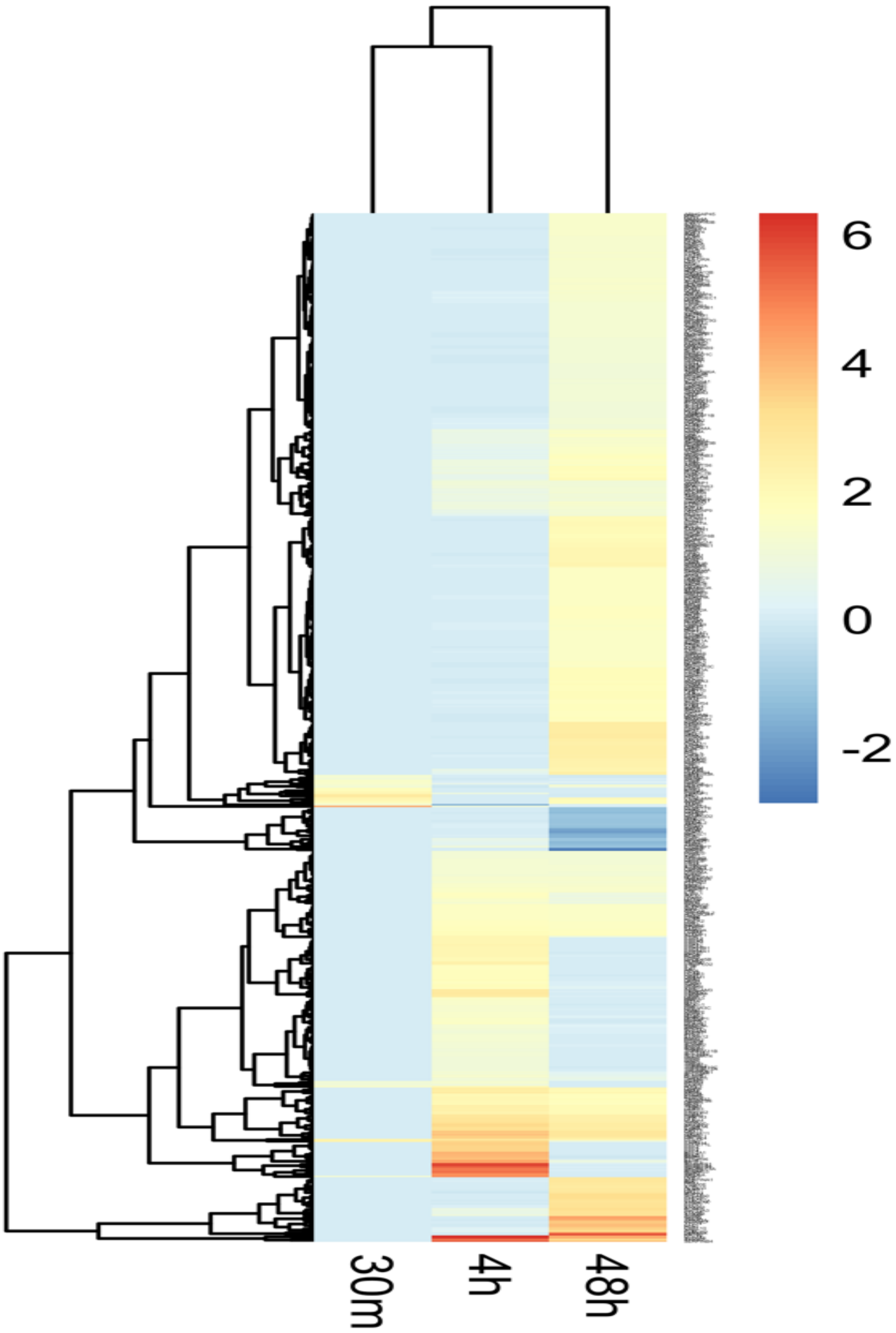

Supplementary Figure 2**. Comprehensive heat map of all differentially expressed genes at all time points**. Gene expression in dissociated skin at 30 minutes, 4 hours and 48 hours after mosquito bite are either significantly upregulated (red) or downregulated (blue).

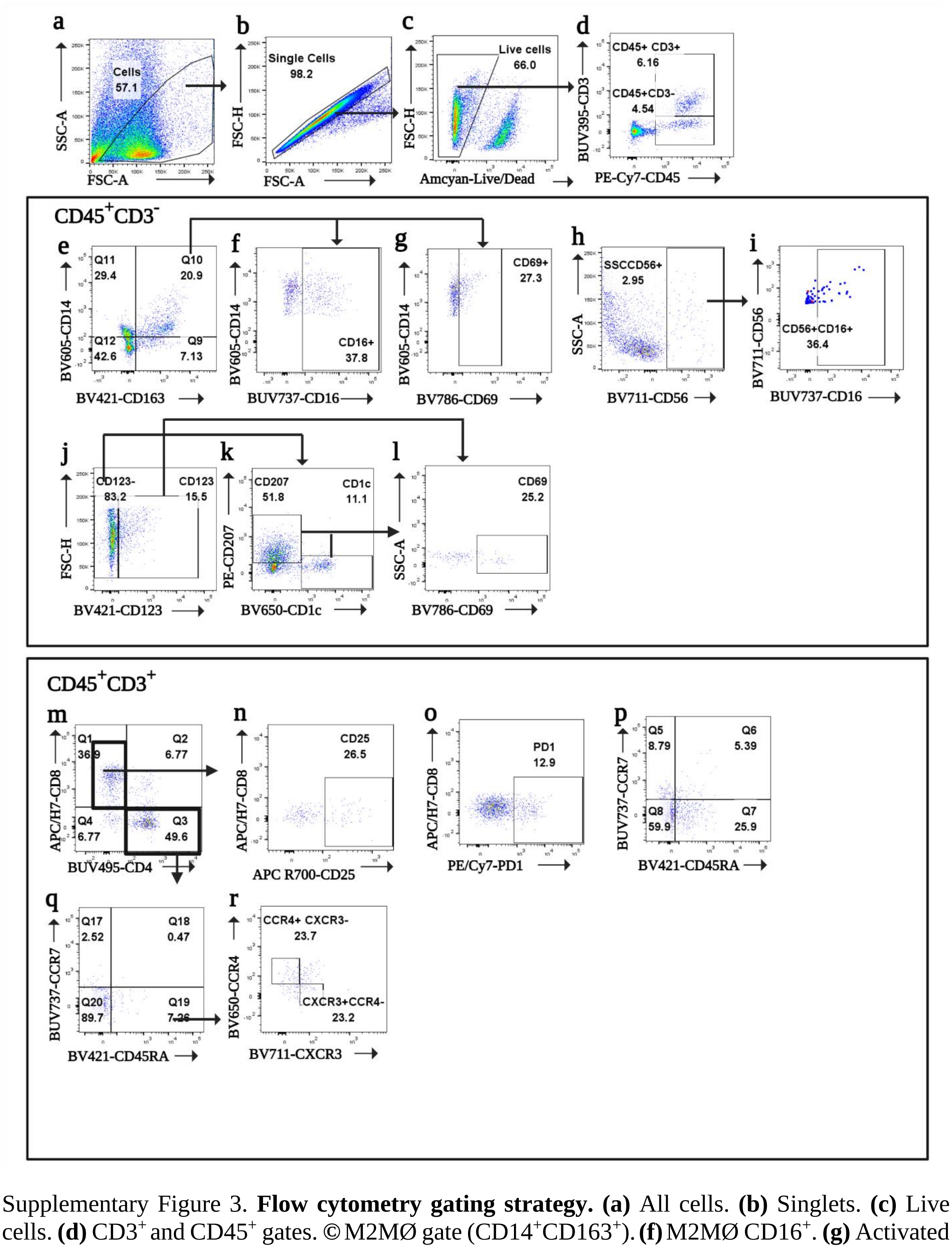

Supplementary Figure 3. **Flow cytometry gating strategy.** **(a)** All cells. **(b)** Singlets. **(c)** Live cells. **(d)** $CD3^+$ and $CD45^+$ gates. © M2MØ gate ($CD14^+CD163^+$). **(f)** M2MØ $CD16^+$. **(g)** Activated M2MØ. **(h)** NK cells. **(i)** NK cells $CD16^+$. **(j)** Plasmacytoid DCs. **(k)** Langerhans cells ($CD207^+$) dermal DCs ($CD1c^+$). **(l)** Activated Langerhans cells/dermal DCs. **(m)** $CD8^+$ and $CD4^+$ T cells

gates. **(n)** Activated CD8$^+$ T cells. **(o)** PD1$^+$ CD8$^+$ T cells. **(p- q)** Central memory (CCR7$^+$CD45RA$^-$), Naïve (CCR7$^+$CD45RA$^+$), Effector memory (CCR7$^-$CD45RA$^-$) and Terminal differentiated effector memory (CCR7$^-$CD45RA$^+$). **(r)** Th17/Th2 (CCR4$^+$CXCR3$^-$) and Th17/Th1 (CCR4$^-$CXCR3$^+$) compartments.

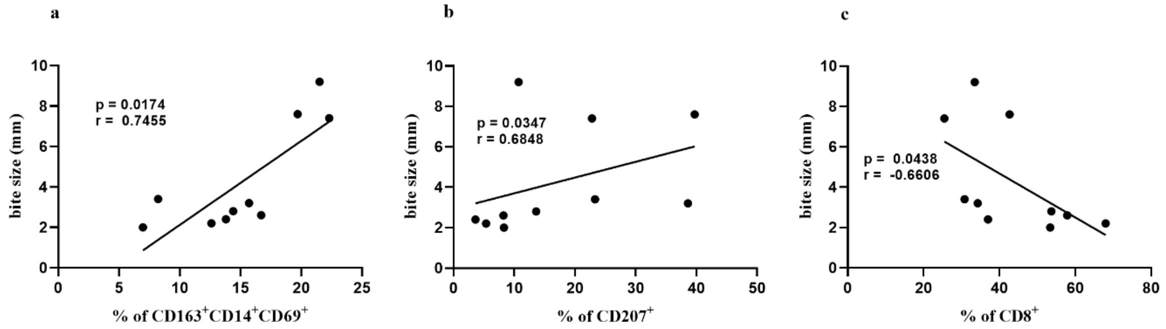

Supplementary Figure 4. **Clinically observed changes in 'bite size' correlate to various immunological changes. (a)** Positive correlation between bite size at 30 minutes and frequency of activated M2 macrophages (CD163$^+$CD14$^+$CD69$^+$) and Langerhans cells **(b). (c)** Negative correlation between bite size at 30 minutes and frequency of CD8+ T cells at 48 hours. Statistical analysis was performed with Pearson correlation coefficient, two tailed. N=10 individuals. Source data are provided as a Source Data file.

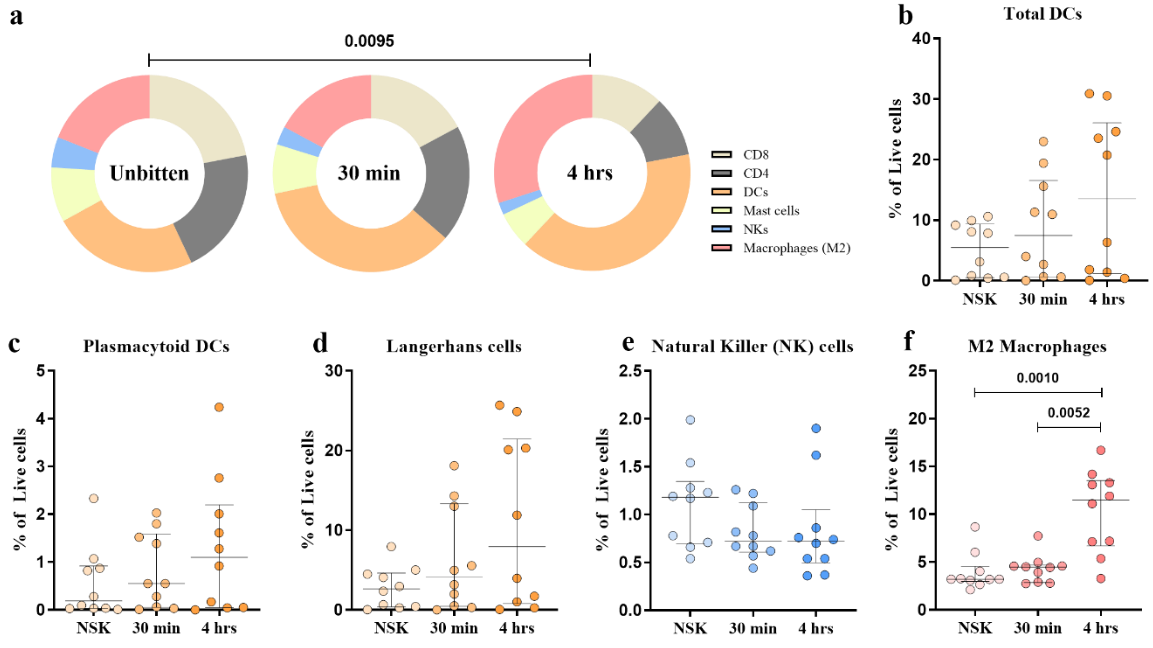

Supplementary Figure 5. **Innate immune response to mosquito bites via flow cytometric analyses.** **(a)** Pie charts showing changes in the frequencies of skin immune cells during early innate immune response to mosquito bite at 30 minutes and 4 hours after exposure. **(b-f)** Frequencies are reported as percentages of total live cells. Statistical analysis was performed

with Chi-square test, two tailed (a) and Friedman + Dunn's multiple comparisons test, two tailed with adjusted p values reported (b-f). Bars indicate median and interquartile range. N=10 individuals. Only p values <0.05 are reported. Source data are provided as a Source Data file.

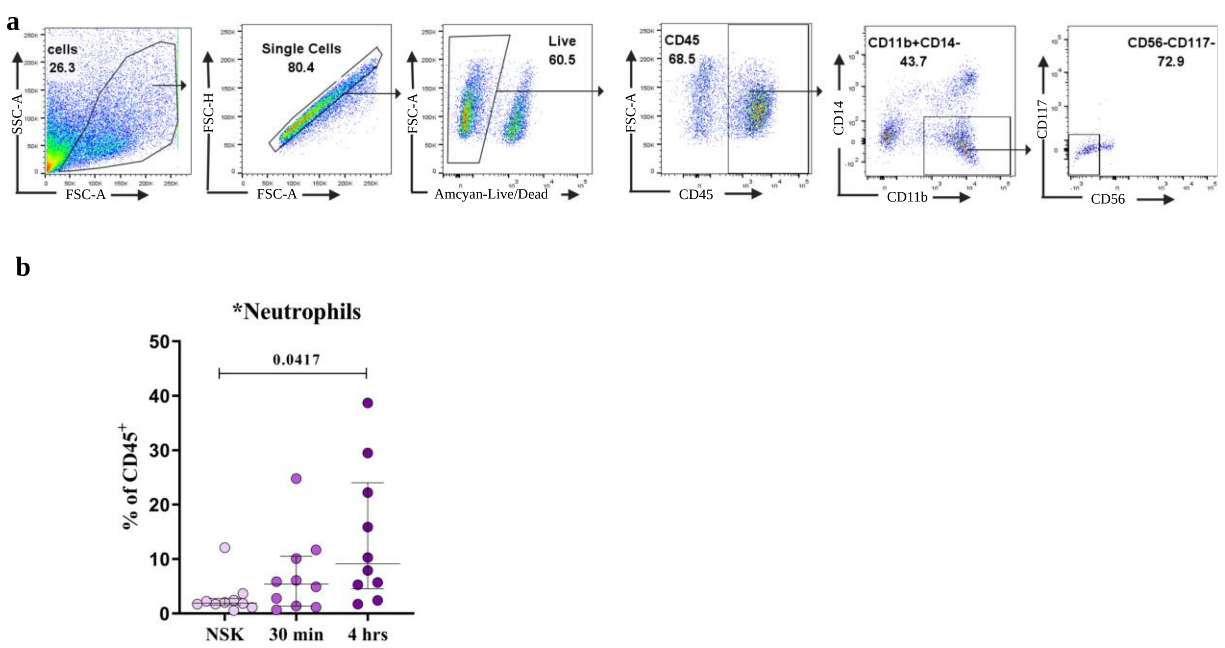

***Defined as CD45^{+}CD11b^{+}CD14^{-} CD56^{-}CD117^{-}**

Supplementary Figure 6. **Changes in myeloid cells CD45^{+}CD11b^{+}CD14^{-}CD56^{-}CD117^{-} frequencies. (a)** Gating strategy defining the population of myeloid cells excluding lymphocytes, monocytes/macrophages, NK cells and mast cells. **(b)** Change in the frequency of neutrophils – defined as CD45^{+}CD11b^{+}CD14^{-}CD56^{-}CD117^{-}. Statistical analyses were performed with Friedman + Dunn's multiple comparisons test, two tailed with adjusted p-values reported. Bars indicate median and interquartile range. N=10 individuals. Only p values <0.05 are reported. Source data are provided as a Source Data file.

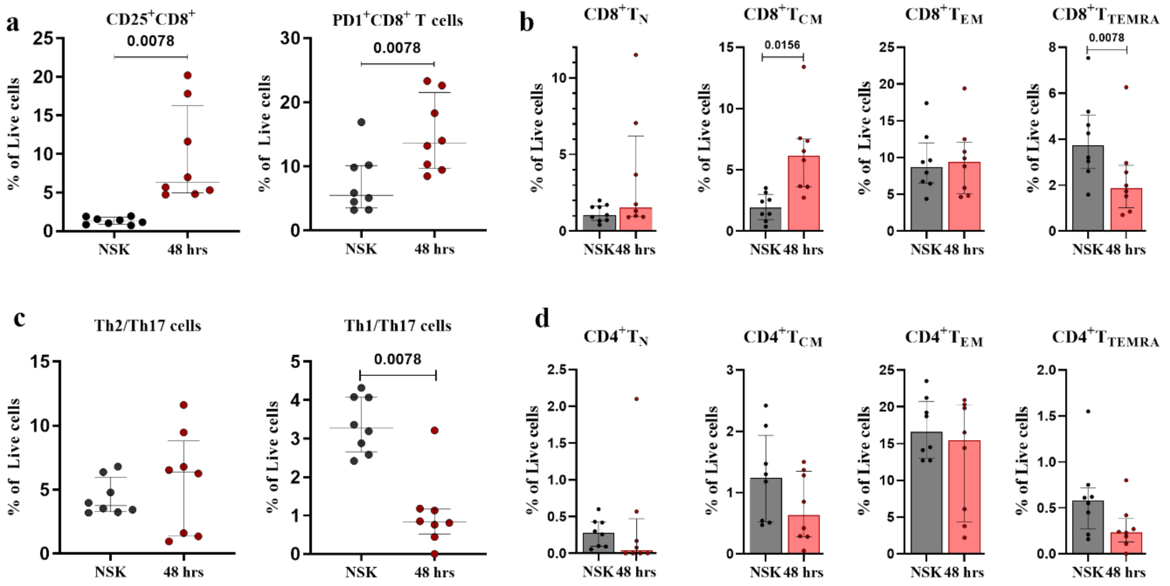

Supplementary Figure 7. **Changes in frequencies of CD4^{+} and CD8^{+} T cell populations as percentages of total live cells.** Statistical analysis was performed with Wilcoxon signed-rank test, two tailed (a-d). Bars indicate median and interquartile range. N=8 individuals. Only p values <0.05 are reported. Source data are provided as a Source Data file.

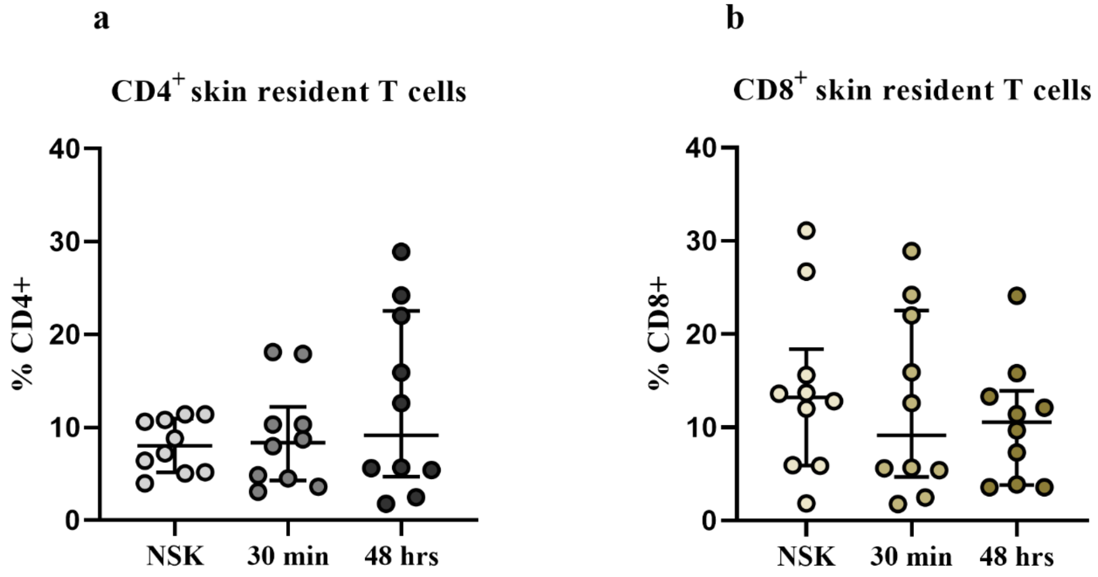

Supplementary Figure 8. **No changes are observed in the frequencies of resident T cells at any time point post mosquito bite. (a)** Frequencies of CD4^{+}CD103^{+}CLA^{+} and **(b)** CD8^{+}CD103^{+}CLA^{+} T cells in normal skin and from biopsies at 30 minutes and 4 hours post-bite. Statistical analyses were performed with Friedman + Dunn's multiple comparisons test, two tailed.

Bars indicate median and interquartile range. Only p values <0.05 are reported N=10 individuals. Source data are provided as a Source Data file.

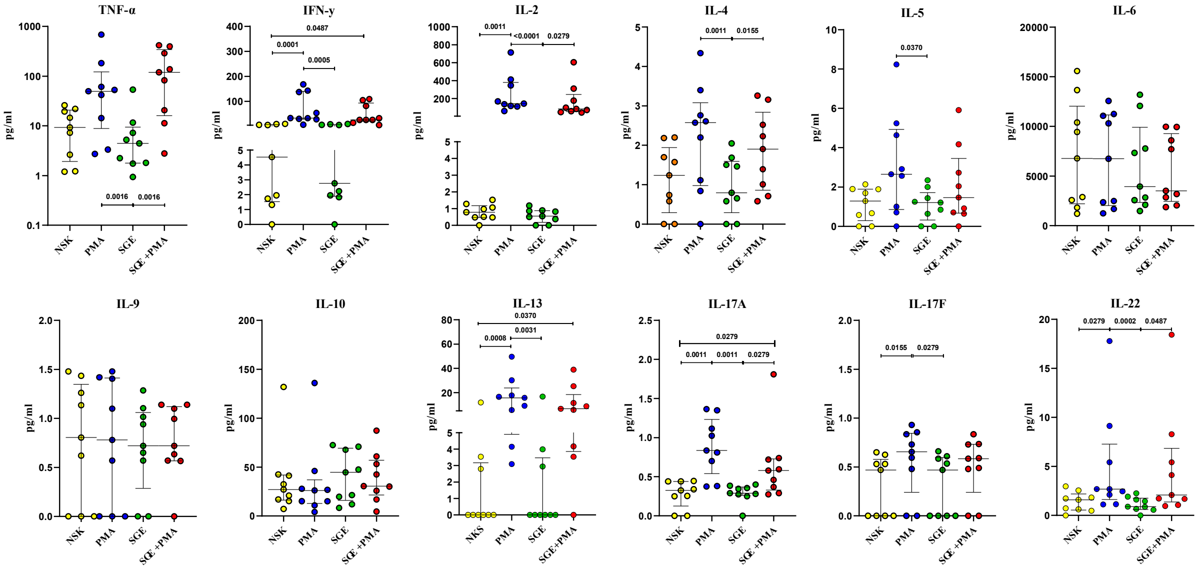

Supplementary Figure 9. **Cytokine production after stimulation with PMA/Ionomycin preparation.** Cytokine production measured in skin cell culture supernatant after treatment with PMA/Ionomycin in the presence or absence of SGE. Skin dissociated cells were seeded on round bottom 96-well plates (50.000 cells/well) and treated with SGE (10 µg/mL) or PBS for 24 hours. Cells were stimulated with PMA (0.1µg/mL) and Ionomycin (1µg/mL) or left unstimulated for the last 6 hours of the culture. Statistical analysis was performed with Friedman + Dunn's multiple comparisons test, two tailed with adjusted p-values reported. Bars indicate median an interquartile range. N=9 individuals. Only p values <0.05 are reported. Source data are provided as a Source Data file.
