# Supplementary material for: Evaluation of cutaneous immune response in a controlled human in vivo model of mosquito bites
Source: Nat Commun. 2022 Nov 17;13:7036. doi: 10.1038/s41467-022-34534-9 (PMC9672097; doi:10.1038/s41467-022-34534-9)
Supplement: Supplementary file 3 — Description of Additional Supplementary Files [file 41467_2022_34534_MOESM3_ESM.pdf]

Description of Additional Supplementary Files

Supplementary Data 1. List of differentially expressed genes in excel format.

Supplementary Data 2. List of over represented pathways at the Reactome database in excel format.

Supplementary Data 3. DEG under an FDR cutoff <0.05 and log2FC >1 in at least one timepoint in excel format
