# Supplementary material for: Evaluation of cutaneous immune response in a controlled human in vivo model of mosquito bites
Source: Nat Commun. 2022 Nov 17;13:7036. doi: 10.1038/s41467-022-34534-9 (PMC9672097; doi:10.1038/s41467-022-34534-9)
Supplement: Supplementary file 7 — Reporting Summary [file 41467_2022_34534_MOESM7_ESM.pdf]

nature portfolio

Corresponding author(s): Jessica E. Manning

Last updated by author(s): Oct 11, 2022

Reporting Summary

Nature Portfolio wishes to improve the reproducibility of the work that we publish. This form provides structure for consistency and transparency in reporting. For further information on Nature Portfolio policies, see our Editorial Policies and the Editorial Policy Checklist.

Statistics

For all statistical analyses, confirm that the following items are present in the figure legend, table legend, main text, or Methods section.

n/a	Confirmed	
☐	☒	The exact sample size (*n*) for each experimental group/condition, given as a discrete number and unit of measurement
☐	☒	A statement on whether measurements were taken from distinct samples or whether the same sample was measured repeatedly
☐	☒	The statistical test(s) used AND whether they are one- or two-sided<br>*Only common tests should be described solely by name; describe more complex techniques in the Methods section.*
☒	☐	A description of all covariates tested
☐	☒	A description of any assumptions or corrections, such as tests of normality and adjustment for multiple comparisons
☐	☒	A full description of the statistical parameters including central tendency (e.g. means) or other basic estimates (e.g. regression coefficient) AND variation (e.g. standard deviation) or associated estimates of uncertainty (e.g. confidence intervals)
☐	☒	For null hypothesis testing, the test statistic (e.g. F, t, r) with confidence intervals, effect sizes, degrees of freedom and P value noted<br>*Give P values as exact values whenever suitable.*
☒	☐	For Bayesian analysis, information on the choice of priors and Markov chain Monte Carlo settings
☒	☐	For hierarchical and complex designs, identification of the appropriate level for tests and full reporting of outcomes
☐	☒	Estimates of effect sizes (e.g. Cohen's *d*, Pearson's *r*), indicating how they were calculated

Our web collection on statistics for biologists contains articles on many of the points above.

Software and code

Policy information about availability of computer code

Data collection	BD FACSAria Fusion: Flow cytometry data was collected using a BD FACSDiva software v9.0 (BD Biosiences).<br>BD FACSCanto II: Data from the multiplex assay was collected using a BD FACSDiva software v8.0.1 (BD Biosiences).<br>NovaSeq 6000 (Illumina): Sequencing of RNA libraries was done using a NovaSeq 6000 from Illumina.<br>RSEM/SAMtools: mapping to the human genome and gene-level quantification were generated by RSEM and SAMtools<br>Microtiter plate reader (BioRad Xmark): IgG ELISA data was collected using a microtiter plate reader.
Data analysis	Statistical analysis and graph design for flow cytometry analysis was done using Prism 9 (Graphpad USA)<br>Cytokine analysis was done using LEGENDplex software v8.0 (Biolegend)<br>Flow cytometry analysis was performed using FlowJo software V10.8.1 (BD Biosciences)<br>Normalization and differential gene expression (DGE) analysis were performed using DESeq2 1.34.0 (Bioconductor)

For manuscripts utilizing custom algorithms or software that are central to the research but not yet described in published literature, software must be made available to editors and reviewers. We strongly encourage code deposition in a community repository (e.g. GitHub). See the Nature Portfolio guidelines for submitting code & software for further information.

Data

Policy information about availability of data

All manuscripts must include a data availability statement. This statement should provide the following information, where applicable:

- Accession codes, unique identifiers, or web links for publicly available datasets
- A description of any restrictions on data availability
- For clinical datasets or third party data, please ensure that the statement adheres to our policy

The Illumina raw reads (fastq files) were loaded at the NCBI Sequence Read Archive with accession numbers SRR18215008 to SRR18215047 and deposited in GenBank under bioproject accession code PRJNA812009. All data generated or analyzed during this study are included in this published article (and its supplementary information files). Clinical trial data is available at https://clinicaltrials.gov/ct2/show/NCT04350905. Flow cytometry data and cytokine data are available now in source data file.

Human research participants

Policy information about studies involving human research participants and Sex and Gender in Research.

Reporting on sex and gender	Biological attributes of sex were collected and reported in Table 1 under the #,frequency of female. All Cambodians participants were cis gendered.
Population characteristics	The study population comprised 13 females and 17 males, all of whom were of Khmer (Cambodian) ethnicity. Age, median with interquartile range was 32.5 [21.75, 37]. 28 of the 30 subjects reported having experienced mosquito bites in the past 30 days before biopsy collection. Past dengue infection was self reported in 3 of the 30 participants.
Recruitment	Participants were recruited by word-of-mouth in Chbar Mon, Kampong Speu, Cambodia. The primary bias may be that most of these individuals were unemployed given the study procedures were performed during the workweek/workday, which would have made other employed individuals less available for recruitment.
Ethics oversight	National Institutes of Health Institutional Review Board and the National Ethics Council on Human Research (Cambodia)

Note that full information on the approval of the study protocol must also be provided in the manuscript.

Field-specific reporting

Please select the one below that is the best fit for your research. If you are not sure, read the appropriate sections before making your selection.

☒ Life sciences ☐ Behavioural & social sciences ☐ Ecological, evolutionary & environmental sciences

For a reference copy of the document with all sections, see nature.com/documents/nr-reporting-summary-flat.pdf

Life sciences study design

All studies must disclose on these points even when the disclosure is negative.

Sample size	Feasibility and sample sizes are adapted from literature concerning neoplastic or autoimmune disorders of the skin versus healthy comparator skin. With regard to flow cytometry of skin samples, 4-mm biopsies typically yield 2200 ±615 cells in healthy skin and 178,000 cells in lesional skin of psoriatic patients. Previous studies of skin transcriptomes have estimated approximately 13,000 genes per biopsy of healthy individuals.
Data exclusions	Exclusion criteria were pre-established as listed on clinicaltrials.gov. Patient samples with insufficient cell counts as determined by flow cytometry were excluded from the study. Samples with less than 1500 total CD45+ cells were excluded
Replication	Experiments on human participants undergoing an invasive procedure as the one performed in this study cannot easily be replicated due to limited availability of patient biological material. Nevertheless, all experiments were standardized and performed before initiation of the study to assure reproducibility, all attempts at replication were successful.
Randomization	Samples were allocated randomly into experimental groups
Blinding	Investigators were blinded to group allocations during data collection and analysis of sample data.

Reporting for specific materials, systems and methods

We require information from authors about some types of materials, experimental systems and methods used in many studies. Here, indicate whether each material, system or method listed is relevant to your study. If you are not sure if a list item applies to your research, read the appropriate section before selecting a response.

Materials & experimental systems

n/a	Involved in the study
☐	☒ Antibodies
☒	☐ Eukaryotic cell lines
☒	☐ Palaeontology and archaeology
☐	☒ Animals and other organisms
☐	☒ Clinical data
☒	☐ Dual use research of concern

Methods

n/a	Involved in the study
☒	☐ ChIP-seq
☐	☒ Flow cytometry
☒	☐ MRI-based neuroimaging

Antibodies

Antibodies used

BUV395 Mouse Anti-Human CD3 (BD biosciences, cat# 564001, Clone SK7, Lot#0030938)
BUV496 Mouse Anti-Human CD4 (BD biosciences, cat# 612936, Clone SK3, Lot#0346317)
BUV737 Mouse Anti-Human CCR7 (CD197) (BD biosciences, cat# 749676, Clone 2-L1-A, Lot#1025804)
APC-H7 Mouse anti-Human CD8 (BD biosciences, cat# 560179, Clone SK1, Lot#1039448)
APC-R700 Mouse Anti-Human CD25 (BD biosciences, cat# 565106, Clone 2A3, Lot#0238004)
Brilliant Violet 785™ anti-human CD69 (BioLegend, cat# 310932, Clone FN50, Lot#B326498)
Brilliant Violet 421™ anti-human CD45RA (BioLegend, cat# 304130, Clone HI100, Lot#B333212)
Brilliant Violet 711™ anti-human CD183 (CXCR3) (BioLegend, cat# 353732, Clone G025H7, Lot#B264427)
PE Mouse anti-human CD207 (Langerin) (BioLegend, cat# 352203, Clone 10E2, Lot#B253018)
Brilliant Violet 650™ Mouse anti-human CD1c (BioLegend, cat# 331541, Clone L161, Lot#B297623)
BB515 Mouse Anti-Human CD117 (BD biosciences, cat# 565172, Clone 104D2, Lot#0119993)
Brilliant Violet 711™ Mouse anti-human CD56 (NCAM) (BioLegend, cat# 331541, Clone 5.1H11, Lot#B318236)
Brilliant Violet 605™ Mouse anti-human CD14 (BioLegend, cat# 367125, Clone 63D3, Lot#B307957)
Brilliant Violet 421™ Mouse anti-human CD163 (BioLegend, cat# 333611, Clone GHI/61, Lot#B315613)
BB700 Mouse Anti-Human CD11b (BD biosciences, cat# 746004, Clone D12, Lot#0239883)
BUV737 Mouse Anti-Human CD16 (BD biosciences, cat# 612787, Clone 3G8, Lot#1198885)
BV650 Mouse Anti-Human CD194 (BD biosciences, cat# 744140, Clone 1G1, Lot#0239874)
PE-Cy™7 Mouse anti-Human CD279 (PD-1) (BD biosciences, cat# 561272, Clone EH12.1, Lot#0022949)
BV605 Rat Anti-Human Cutaneous Lymphocyte Antigen CLA (BD biosciences, cat# 563960, Clone HECA-452, Lot#0177315)
PE/Dazzle™ 594 anti-human CD45 (BioLegend, cat# 982308, Clone HI30, Lot#B350011)
goat anti-human IgG alkaline phosphatase (Millipore-Sigma; Cat #AP113A; Lot 029M4838V)
Dengue IGG (PanBio; CAT 01PE30; Lot 01P30F005)

Validation

As stated in the manufacturer's website:

BD Biosciences "The production process underwent stringent testing and validation to assure that it generates a high-quality conjugate with consistent performance and specific binding activity. However, verification testing has not been performed on all conjugate lots"

Biolegend's website provides a searching tool, where query on specific lot numbers can be done for a certificate of analysis and quality control of each antibody.

For detailed information on the validation procedures please follow the links to the manufacturer's websites:

https://www.bdbiosciences.com/en-eu/products/reagents/flow-cytometry-reagents

https://www.biolegend.com/de-at/products

Animals and other research organisms

Policy information about studies involving animals; ARRIVE guidelines recommended for reporting animal research, and Sex and Gender in Research

Laboratory animals

For laboratory animals, report species, strain and age OR state that the study did not involve laboratory animals.

Wild animals

Field collected Aedes larvae were hatched, speciated, and Aedes aegypti mosquitos were reared to adulthood in an ACL-2 insectary under typical conditions. The F3 and F4 generations were tested for circulating arboviruses via PCR (dengue, chikungunya, Zika) and were negative. The F5 generation was then hatched and kept separate from the colony for human use. They fed on nectar water and received no blood meals until they were 8-10 days of age and could successfully take a bloodmeal on human participants in a controlled manner. The mosquitos were sacrificed afterwards by being placed in a -80C freezer and then incinerated.

Reporting on sex

Field-collected samples

For laboratory work with field-collected samples, describe all relevant parameters such as housing, maintenance, temperature,

Field-collected samples | *photoperiod and end-of-experiment protocol OR state that the study did not involve samples collected from the field.*

Ethics oversight | *Identify the organization(s) that approved or provided guidance on the study protocol, OR state that no ethical approval or guidance was required and explain why not.*

Note that full information on the approval of the study protocol must also be provided in the manuscript.

Clinical data

Policy information about clinical studies
All manuscripts should comply with the ICMJE guidelines for publication of clinical research and a completed CONSORT checklist must be included with all submissions.

Clinical trial registration: NCT04350905

Study protocol: clinicaltrials.gov

Data collection: From October 2020 to January 2021, data and samples were collected at Kampong Speu District Referral Hospital, Cambodia

Outcomes:
The primary outcomes as defined in the study protocol are:
1. Measurement of changes in the early and late innate immune response and cellular recruitment in bitten skin versus unbitten skin by:
a. immunophenotyping of innate immune cell subsets in dissociated skin sample at Day 0 timepoints
b. determination of cytokine profile in dissociated skin sample supernatant at Day 0 timepoints
c. differential complementary DNA (cDNA) expression prepared from skin RNA and analyzed via RNA sequencing (RNASeq) at Day 0 timepoints
2. Measurement of changes in the adaptive immune response and cellular recruitment bitten skin versus unbitten skin by:
a. phenotyping of adaptive immune cell subsets in dissociated skin sample at Day 2 timepoints
b. determination of cytokine profile in dissociated skin sample supernatant at Day 2 timepoints
c. differential cDNA expression prepared from skin RNA and analyzed via RNASeq at Day 2 timepoints

Flow Cytometry

Plots

Confirm that:

☒ The axis labels state the marker and fluorochrome used (e.g. CD4-FITC).

☒ The axis scales are clearly visible. Include numbers along axes only for bottom left plot of group (a 'group' is an analysis of identical markers).

☒ All plots are contour plots with outliers or pseudocolor plots.

☒ A numerical value for number of cells or percentage (with statistics) is provided.

Methodology

Sample preparation: Whole skin dissociation kit (Miltenyi®) was used to perform the biopsies dissociation. An enzyme mix consisting of enzyme D, enzyme A and buffer L was prepared according to the manufacturer's specifications. Single punch biopsies were incubated in 400 µl of dissociation mix, at 37°C – 5% CO2 overnight. Following incubation, each sample mix was transferred to a Falcon round-bottom tube with cell strainer cap (40-75um). Using a rubber pestle and the cell-strainer cap as a mortar, biopsies where gently macerated to increase cell recovery yield. 500 µl of cold RMPI was used to rinse caps. Samples were then centrifuged at 428 x g at 4°C for 10 minutes and the pellet resuspended in 500 µl of cold RPMI. Dissociated skin cells were counted using a pre-programmed setting in a Countess™ II FL Automated Cell Counter.

Instrument: BD FACSAria Fusion (BD Biosciences)

Software:
Acquisition: BD FACSDiva software (BD Bioseceinces)
Analysis: FlowJo X 10.0.7r2 software.

Cell population abundance: Patient's dissociated skin: on average 60.000 dissociated cells were analyzed per patient for each panel. Total cell counts for patient samples were variable. In resting skin the distribution of the different cell types analyzed was as on average: T cells (42%), DCs (24%), Mast cells (10%), NK cells (5%) and Macrophages (19%).

Populations distribution changed at the different time points, as presented in the manuscript.

Gating strategy: Total cells were gated in a SSC-A vs FSC-A plot and then filtered through a gate for single cells using a FSC-H vs FSC-A

Gating strategy

configuration. Viable cells where separated from dead cells using a viability stain and then plotted using a CD3 vs CD45 marker combination to divide lymphocytes and the rest of the leukocytes.

Macrophages were gated from CD14 vs CD163 plots as the double positive populations. In these populations new gates for CD16 and CD69 markers were created in order to analyze the expression of these activation markers.

NK cells were identified in plots using a CD56 marker. From the CD56+ populations we created gates for CD16 marker to identify cytotoxic NK cells.

Plasmacytoid populations were gated using a CD123 marker. From the positive population, CD69 gates were created to analyze activation. From the CD123- population, CD207 vs CD1c plots were created to identify Langerhans and dermal dendritic cell populations. CD69 gates were also applied to these populations to determine activation.

T cells were gated from CD8 vs CD4 plots. For both CD8 and CD4 populations, CD25 and PD1 gates were created to observe activated subpopulations and CCR7 vs CD45RA plots to analyze memory subsets (Naive CCR7+CD45RA+, central memory CCR7+CD45RA-, effector memory CCR7-CD45RA- and terminal differentiated effector CCR7-CD45RA+). An additional CCR4 vs CXCR3 plot was created for Naive CD4 populations in order to determine the proportions of Th1/Th17 and Th2/Th17 subpopulations.

☒ Tick this box to confirm that a figure exemplifying the gating strategy is provided in the Supplementary Information.
